# Co-Occurrence of *Helicobacter pylori* and *Candida* spp. Infections in the Pathogenesis of Gastrointestinal Diseases

**DOI:** 10.3390/biomedicines13051172

**Published:** 2025-05-11

**Authors:** Joanna Braksator, Anna Kofla-Dłubacz, Katarzyna Antosz-Popiołek, Hubert Szyller, Joanna Koga-Batko, Martyna Wrześniewska, Maciej Dyda, Tomasz Pytrus

**Affiliations:** 1 2nd Clinical Department of Paediatrics, Gastroenterology and Nutrition, Wroclaw Medical University, 50-369 Wroclaw, Poland; joanna.braksator@umw.edu.pl (J.B.); anna.kofla-dlubacz@umw.edu.pl (A.K.-D.); tomasz.pytrus@umw.edu.pl (T.P.); 2 Student Scientific Group of Pediatric Gastroenterology and Nutrition, Wroclaw Medical University, 50-369 Wroclaw, Poland; antosz0715@gmail.com (K.A.-P.); joanna.batko99@gmail.com (J.K.-B.); martyna.wrzesniewska@gmail.com (M.W.);

**Keywords:** *Helicobacter pylori*, candida, eradication, microbial interactions, gastrointestinal microbiota

## Abstract

*Helicobacter pylori* and *Candida* spp. are widespread microorganisms found in the human gastrointestinal tract, often coexisting in the same ecological niche. *H. pylori*, a Gram-negative bacterium, is a well-known pathogen responsible for gastritis, peptic ulcers, and gastric cancer. In contrast, *Candida* fungi, often detected in food, particularly *Candida albicans*, are generally considered commensal organisms, but can become opportunistic pathogens under certain conditions. Recent studies suggest a possible link between these microorganisms, highlighting a new survival strategy of *H. pylori*, that is, its ability to internalize in *Candida* vacuoles. This phenomenon, confirmed by various microscopic and molecular techniques, may provide *H. pylori* with protection against adverse environmental conditions, especially clinically important antibiotic therapy. The basic premise of this theory is the ability of *H. pylori* to penetrate vacuoles in fungal cells, which then become a reservoir of infection, allowing the infection to recur. Understanding the interaction between *H. pylori* and *Candida* may offer new insights into the pathogenesis of gastrointestinal diseases and may lead to the development of treatments targeting both organisms simultaneously. The purpose of this article is to review the literature, considering the first observations on this problem in the literature and the current state of knowledge, and to suggest a direction for further research.

## 1. Introduction

Gastrointestinal infections are a significant clinical and epidemiological problem, with their etiology increasingly recognized as multifactorial, emphasizing the interactions between bacteria, fungi, and the host’s immune system. In recent years, there has been growing interest in the possible interaction between *H. pylori* and *Candida* spp., especially focusing on *C. albicans* which is often considered as a common component of the natural human flora. Those studies have shown that *H. pylori* may survive in the vacuoles of *Candida* spp. and use them as protective niches, which protects the bacterium from difficult environmental conditions [1]. This paper aims to explore the co-occurrence of both microorganisms in the context of their involvement in the pathogenesis of gastrointestinal diseases. Understanding the interactions between them could offer new insights into future therapeutic and preventive strategies. Over 36% of patients with gastric ulcers, and over 56% of patients with large gastric ulcers (more than 2 cm) had fungal co-colonization with *H. pylori* of the upper gastrointestinal (GI) tract, suggesting a close relationship between *Candida* spp. and *H. pylori* in peptic ulcers, but also in gastric cancer (GC) and chronic gastritis [2]. Starting the discussion about the connection between *H. pylori* and *Candida albicans* requires their introduction and presentation.

This paper is an attempt to compile the current state of knowledge on the intracellular presence of *H. pylori* in Candida cells present in the human gastrointestinal tract and to familiarize physicians with the problems associated with the eradication of *H. pylori*. The article’s main aim is to explore the co-occurrence and potential synergistic interactions between *H. pylori* and *Candida* spp., particularly *C. albicans*, in the pathogenesis of gastrointestinal diseases. By analyzing the intracellular presence of *H. pylori* within *Candida* vacuoles and its implications for treatment resistance and disease severity, we seek to highlight novel mechanisms contributing to infection persistence and therapeutic challenges.

## 2. Methodology

In principle, the process of searching for publications on a given topic was in line with the assumptions of the Preferred Reporting Items for Systematic Reviews and Meta-Analyses (PRISMA), although due to the niche topic and the significant research in the debate from different periods of time, the assumptions had to be partially modified.

Electronic database searches focused firstly on original articles, meta-analyses, and systematic reviews. The databases were searched using PubMed. The research included a review of work from the last 10 years up to March 2025. Additionally, significant works directly related to the subject of the thesis and from a different time period were also selected.

The keywords included terms such as *Helicobacter pylori*; Candida; eradication; microbial interactions; gastrointestinal microbiota. The keywords were used both in combination and separately.

Articles in languages other than English, not directly related to the topic described, and outdated or too limited were excluded. In exceptional cases, works written in Polish were also cited due to the origin of the authors and the possibility of referring in the work to research written in their region.

## 3. *Helicobacter pylori*

*H. pylori* is a Gram-negative microaerophilic, ubiquitous bacteria that can change from spiral to coccoid, which inhabits the human stomach niche, is the most abundant microbe included in the microbiota of the gastric cavity, discovered for the first time by Barry J. Marshall and Robin Warren in 1982, later awarded with the Nobel Prize in 2005 [3,4,5]. This revolutionary research allowed the development of effective treatment for diseases of previously unknown etiology [3].

*H. pylori* presence is primarily associated with several gastrointestinal diseases, of which the most common are chronic gastritis and gastric and duodenal ulcers. Furthermore, *H. pylori* infection is the most significant cause of stomach adenocarcinoma, the sixth most diagnosed cancer in the world, and lymphoid tissue lymphoma (MALT lymphoma) [3,4,5,6,7,8]. Although at first most patients are asymptomatic, most of them will develop gastrointestinal symptoms caused by long-lasting *H. pylori* infection [8].

The link between infection with this bacterium and diseases such as idiopathic thrombocytopenic purpura, sideropenic anemia, and vitamin B12 has been widely studied [9] and currently, there is a progressively increasing interest in its connection to the development of cardiovascular, metabolic, and neurologic disorders, including neurodegenerative diseases [10,11,12,13].

*Helicobacter pylori* uses its flagella to penetrate the protective mucus layer and adhere to the epithelial surface. Due to the produced urease, it can neutralize the acidic condition from the beginning of infection, creating a microenvironment to survive [14]. Other key virulence factors of *H. pylori* include cytotoxin-associated gene A (CagA) and vacuolating cytotoxin A (VacA). CagA changes the epithelial cell cytoskeleton, affecting the proliferation of cells and stimulating the gastric epithelial cells to secrete interleukin-8. VacA is embedded in the host cell membrane and also has properties of an anion-selective channel that releases bicarbonate and organic anions into the host cytoplasm and supports *H. pylori* colonization by allowing the efflux of potential metabolic substrates for bacterial growth [15].

While *H. pylori* is usually considered an extracellular organism, its facultative intracellular presence by surviving in host cells, such as dendritic cells and macrophages, epithelium or cells in host microbiota, of which the most commonly mentioned are *Candida* yeasts, is demonstrated in an increasing number of studies, which should raise questions not only about the impact of an intracellular presence on the pathogenesis of diseases caused by this microorganism, but should also raise questions about its involvement in failed eradications, which are growing in frequency [16,17,18,19,20]. 

A thorough understanding of the pathogenesis, precise diagnostic approaches, and optimized management strategies for *H. pylori* infections is crucial for minimizing their public health burden. That is why the eradication of *H. pylori* should be given priority as soon as it is diagnosed. To do so, it is necessary to select appropriate therapy, considering local antibiotic resistance and mechanisms that allow bacteria to survive and reinfect. 

## 4. *Candida albicans* and *Candida* spp.

It is worth emphasizing that in this article, when talking about *Candida*, it will very often refer in particular to the *Candida albicans* species, due to its enormous commonness, the amount of available research, as well as the possibilities of applications and interpretation of connections in practice.

*Candida* is classified as a single-cell fungus, specifically speaking, a true yeast, which reproduces by budding. Individual cells are rounded to oval, 3–8 μm in diameter, or filamentous. Usually, both forms are present in affected tissues. *Candida* can be isolated from the surface of the skin, from the mucous membranes of the oral cavity, vagina, and the digestive tract [21,22,23]. It is worth noting that, especially when it comes to *C. albicans,* it is commonly present in the gastric contents of 70% of healthy adults (approximately 10^2^ CFU/mL) [24]. As it is found commonly throughout and inside the human body, it is considered a part of the physiological microbiota. However, the yeasts are considered opportunistic, as they can become pathogenic under specific circumstances, usually when the immune system is impaired by chronic stress, illness, surgery, or specific medications [21,22,23].

Candida produces proteins from the agglutinin-like sequence family (ALS), which is a group of adhesins that adhere to epithelial and endothelial cells and allow yeasts to form a biofilm [25]. Key virulence factors of Candida include aspartyl proteinases (SAPs), phospholipases and lipases, which degrade host proteins, disrupt cell membranes, facilitate tissue invasion, and progress the development of inflammation [26]. Additionally, Candida can stimulate Th17-mediated immune responses and interact with pattern recognition receptors, leading to the production of proinflammatory cytokines (e.g., IL-1β, IL-6, IL-17) [27].

## 5. Epidemiology of *H. pylori* Infections

Currently, infections caused by *H. pylori* constitute a global health problem and its prevalence estimates are summarized in Table 1. 

*H. pylori* infection is predominantly acquired during the early years of life [28]. Socioeconomic status, hygiene, and sanitation are pivotal in determining the risk of *H. pylori* infections [29]. Furthermore, in a study by Smith S. et al., investigating the risk factors for *H. pylori* infection in Nigeria, living with more than three individuals in a household, a family history of ulcers or gastritis, and current use of antibiotics were found to be strongly correlated with *H. pylori* infection [30].

**Table 1:** Worldwide estimates of *H. pylori* prevalence among populations of different world regions, based on [31].

Global Prevalence of *H. pylori*	
Region	Prevalence Estimates, %
African region	56.5
Eastern Mediterranean region	56.5
European region	45.7
Region of the Americas	49.0
Southeast Asia region	44.2
Western Pacific region	49.4

Peptic ulcer disease (PUD) is one of the most common diseases of the gastrointestinal tract. Approximately 90–95% of duodenal ulcers (DU) and 70–75% of gastric ulcers (GU) are associated with the *H. pylori* infection; the lifetime risk for developing peptic ulcer disease among infected patients is around 10% and the concomitance of *H. pylori* infection along with NSAID use raises the risk of bleeding ulcers by more than six times [32,33]. Interestingly, a study by Chen T.-H. et al. showed that with proper PPI and eradication treatment of the *H. pylori*-positive patients, DU prevalence decreased over time while GU prevalence remained unchanged. What is more, among the examined patients with GU, MboI-RFLP-defined genotype 3 was the most prevalent one [34], which suggests a genotypic difference between the GU- and DU-associated *H. pylori* genomes.

*Helicobacter pylori* was graded as a Group I carcinogen in 1994 by the International Agency for Research on Cancer [35]. Its infection is the promoter of Correa’s cancer cascade—a stepwise progression that covers several histological lesions of the gastric mucosa, leading to adenocarcinoma [36]. Recent research has elucidated the mechanisms by which *H. pylori* influences immune responses and tumor progression, particularly through virulence factors such as CagA, oxidative stress, and dysregulated pyroptosis pathways. Additionally, pyroptosis exhibits a dual role in tumor dynamics, and miRNA-1290 has been recognized as a critical regulator and potential biomarker, offering valuable insights for future therapeutic strategies in *H. pylori*-associated gastric cancer [37].

The duration of exposure to *H. pylori* infection translates to an increased risk of gastric cancer. In a retrospective cohort study by Kumar S. et al., it was discovered that the cumulative cancer incidence after *H. pylori* detection was 0.37% at 5 years, 0.5% at 10 years, and 0.65% at 20 years. Increased cancer risk was also linked to older age at detection, certain ethnic groups (black/African American, Asian, Hispanic or Latino), history of smoking, and male sex [38]. Eradication of *H. pylori* has been demonstrated to reverse chronic gastritis in most patients and atrophic gastritis in some cases, though it does not reverse intestinal metaplasia within the gastric cancer cascade [39,40,41]; nevertheless, *H. pylori* eradication has been shown to decelerate the progression of intestinal metaplasia to adenocarcinoma. A 15-year study among 2258 *H. pylori*-seropositive individuals found that one-time treatment significantly reduced gastric cancer incidence and mortality, particularly in those aged 55 and older and in individuals with intestinal metaplasia or dysplasia at baseline [42].

## 6. *H. pylori* Eradication and Its Resistance to Antibiotics

Studies show that common therapies that used to be first-line treatment fail in approximately one in three patients [43]. Choice of treatment is based on local susceptibility to antimicrobials and mainly depends on the level of clarithromycin resistance. This is why testing new therapies and searching for the reasons for *H. pylori* antibiotic resistance is a very important field for scientists [44].

Among the areas with low (<15%) clarithromycin resistance, as Maastricht guidelines recommend, the equivalent first-line treatments are clarithromycin triple and bismuth quadruple therapy. The most used treatment of *H. pylori* infection is still, to this day, the quadruple therapy including bismuth. It generally combines PPI, bismuth, tetracycline and metronidazole. Due to metronidazole resistance, the duration of such treatment should be at least 10 to 14 days [44,45].

The standard triple therapy includes PPI (proton pump inhibitor), clarithromycin, and amoxicillin. The recommended duration of this treatment is 14 days, as it was found that it is more effective than 7- or 10-day-long therapy [44,46]. The main limitation of this approach is the rising resistance of *H. pylori* against clarithromycin [47]. Second-line treatment, if those fail, is triple or quadruple therapy based on PPI, levofloxacin, and amoxicillin, with the addition of bismuth in a quadruple scheme [44]. In regions with high (>15%) or unknown clarithromycin resistance, it is strongly advised to use the quadruple bismuth therapy as a first-line option, and non-bismuth quadruple containing PPI, clarithromycin, amoxicillin, and metronidazole as the first choice only, when the bismuth quadruple is locally unavailable. Second-line therapy is persistent triple or quadruple levofloxacin therapy [44].

According to new *H. pylori* eradication guidelines presented by the American College of Gastroenterology, the first-line therapy is bismuth-based quadruple therapy for 14 days for treatment-naïve patients, and for treatment-experienced patients who failed treatment, should be preceded by an initial course of proton pump inhibitor, clarithromycin, and amoxicillin [48].

It was also found that the PPI used affects the effectiveness of all kinds of treatment. Therefore, it is recommended to use novel PPIs such as esomeprazole, rather than omeprazole, pantoprazole, or lansoprazole [49].

As mentioned before, the main reason for the ineffectiveness of *H. pylori* eradication is its resistance to commonly used antibiotics, which is rising alarmingly over the years [47,50,51,52]. The most alarming are the levels of resistance to metronidazole, clarithromycin, tetracycline, levofloxacin, and amoxicillin [53]. *H. pylori* antibiotic resistance worldwide has been summarized in Table 2. The mechanisms of resistance can be pathogen or host-based. Among others, the pathogen-based mechanisms include the mutation of the cellular target (e.g., genes responsible for protein translation, cell wall synthesis), reduction of intracellular accumulation of antibiotics, biofilm production, production of enzymes inactivating antibiotics, secretion of virulence factors, creating coccoid forms, or inducing autophagy. When it comes to host-based resistance mechanisms, we can observe gene polymorphisms of gastric acid secretion, transport, metabolism, different immune status, or disease background [50,51]. Another mechanism could be the vacuolation of *H. pylori* in *Candida*, which is further expanded in our paper [24,43,52].

## 7. Interplay Between *Candida* spp. and *H. pylori* Infection

Starting from the beginning, in 1978, Katzenstein et al. first detected and confirmed the presence of fungal organisms morphologically resembling Candida spp. in one-third of examined surgically resected gastric ulcers. The presence of fungi in the collected samples was described as an additional risk factor, which can aggravate and perpetuate gastric ulceration [54].

A few years later, another study by Scott et al. (1982) confirmed the presence of Candida in patients with gastric cancer, where the candidiasis appeared to be secondary to structural damage caused by the cancer itself [55]. In addition to these studies, the following year, Warren et al. published a report confirming the presence of Candida in patients with peptic ulcer disease [56].

Two decades later, Zwolińska-Wcisło and her team performed several studies and trials to evaluate the effect of fungal colonization on the course of gastric ulcer disease and chronic gastritis by using an endoscopy of the upper gastrointestinal tract before and following four weeks of standard anti-ulcerous treatment. As a result, Candida colonization has been described as a cause of impaired repair and healing processes in gastric ulcers. The compromised tissues, despite the absence of *H. pylori*, still contributed to the significantly longer duration of ulcer manifestation. Additionally, no correlation between the level of fungal antibodies and the concentration of fungi in the stomach was observed [57,58].

Another significant study valuated the relationship between the presence of Candida and the occurrence of upper gastrointestinal diseases, such as non-ulcer dyspepsia, gastric, and duodenal ulcers. It has been indicated that there is a relationship between the co-occurrence of *H. pylori* and Candida infections, where they act synergistically, causing an increased frequency and severity of upper-tract diseases. As was also noted, the mere presence of fungi, without an accompanying bacterial infection, was not associated with the symptoms of the diseases in question, as these fungi seem to colonize to a clinically insignificant degree (<10^3^ CFU/ml) [59]. 

The above studies focused on the coexistence and synergistic consequences of co-infections. However, the current interest in the co-occurrence of Candida infections with *H. pylori* is not only due to the already described increased gastric damage. Further studies and modernized technologies have shown a closer relationship between these microorganisms.

The starting point of the following considerations is the fact that *H. pylori* features as an invading intracellular pathogen. The fact of entering gastric epithelial cells, dendritic cells, and macrophages by this microorganism is well documented [2,60]. Going further, this is one of the components responsible for difficulties in its eradication. However, there is increasing evidence that *H. pylori* has the ability to invade also much simpler organisms, including the previously mentioned fungus Candida [2,24,61,62].

Before developing this subject, a brief description of the vacuole structures seems necessary, as *H. pylori* potentially inhabits them. Vacuoles are specialized organelles found in plants, some protozoa, and yeast cells. Fungal cells usually contain many small vacuoles. Vacuoles contain numerous ions: sodium, potassium, calcium, chloride, sulfate, nitrogen, and phosphorus, as well as many others, which help maintain a constant level of ions in the cytosol that is safe for the cell’s metabolism [63]. In this context of the discussed issue, such a safe, delimited environment, full of valuable chemical compounds, seems to be a perfect place for a potential escape for *H. pylori*.

**Figure 1:**
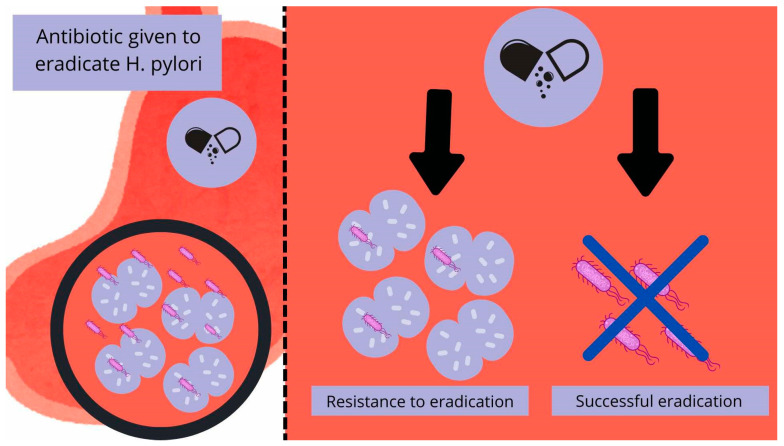
Diagram illustrating the interplay theory between *H. pylori* and *Candida* spp. A gastrointestinal tract infected with *H. pylori* may be resistant to pharmacological eradication due to the use of the fungus’ cell vacuolas as a shelter, enabling subsequent reinfection. The diagram was made by authors.

Initial studies using optical microscopes beginning in 1998 revealed the presence of moving “bacterial-like bodies” (BLBs), seen as dense black dots in a state of movement, in the vacuoles of Candida fungi from the gastric mucosa, which were later identified as *H. pylori* by PCR and immunofluorescence. In numerous studies, these hybrids of “invaded” Candida appear resistant to many harsh environmental conditions, such as high temperature, dryness, and different antibiotics. *H. pylori* inside the vacuole, floating in the yeast cytosol, does not seem to suffer in these tests and remains protected by the fungal structures [1,62,64]. What is more, *H. pylori* appears to not only be able to survive inside the yeasts but to thrive enough that it begins to multiply and spread inside them, by being vertically transmitted between Candida cells, and even expresses its proteins [1]. The numerous studies conducted by Saniee and Siavoshi over many years show the possibilities of internalization and coexistence of these two species. Notably, in the control sample containing free-living bacteria without the help of fungus, the bacteria did not survive the tested conditions [62,64]. The main theoretical assumptions are schematically shown in Figure 1.

Going further into more molecular detail, it seems significant to focus on the issue of research concerning the CagA. This specific gene, which is characteristic for *H. pylori*, was detected in Candida with BLBs. This finding undoubtedly highlights the molecular connection between these two forms. What is more, the CagA gene was also detected in consecutive generations of Candida, which proves that vertical gene transmission of CagA takes place between developing and multiplying yeast [1]. For now, it remains an open question whether these mechanisms act as a protective or a transmitting mechanism, or both. Additionally, this gene appears to be significantly associated with the resistance of vacuolated *H. pylori* to amoxicillin therapy, with the bacteria surviving such treatments in tests. This also leaves the question of resistance to other antibiotics and the possibility of treating such forms [65,66]. Finally, it is worth adding that the epidemiological prevalence of the CagA gene in Western countries is nearly 60% [15] and it is a gene potentially responsible for a more severe course of *H. pylori* infection, resulting in the development of peptic ulcer disease, compared to patients infected with CagA (-) forms [15,59].

It is important to take into consideration that Candida occurs not only in human organisms, but it is also known that some strains can be found in food products such as milk, yogurt, bread, fruit, honey, and juice. It was found that some of them can carry *H. pylori* in their vacuoles [67]. Thus, Candida present in such products can play a vital role in the transmission of *H. pylori*, mainly because, as mentioned before, it was found that the sequestration in yeast vacuoles enhances *H. pylori* survival in unfavorable conditions, including an acidic pH or anaerobic environment [18,24,64,68,69]. Some studies also showed that the internalization of *H. pylori* into Candida can be triggered by certain antibiotics, mainly amoxicillin, which may result in the ineffectiveness of treatment that includes it. Candida can also be resistant to high temperatures and other technological processes used in food processing, which may enable another way of transmission of *H. pylori* by yeast [70]. The vacuoles of Candida can store glycoproteins, Ca^2+^, amino acids, phosphoranes, whereas their cell membrane is rich in ergosterol, which plays a vital role in the colonization of human organisms [71]. All the mentioned properties of Candida make it a perfect environment and vector for *H. pylori*. A study conducted on mice model by Hiengrach et al. showed that inflammation scores and the levels of gastric TNF-α, IL-6, and IL-10 were lower among the group of mice models infected with *Candida* containing *H. pylori*, compared to mice infected with *H. pylori* alone. However, the severity of epithelial defects was significantly greater in the group containing *Candida*-associated *H. pylori*. All of the differences were statistically significant. This study also emphasized that amoxicillin was ineffective against *H. pylori* associated with Candida, despite the strain being sensitive to both low and high concentrations of the antibiotic under aerobic conditions. This is most likely connected to the antibiotic’s inability to penetrate or reduce the Candida colonies. The intravacuolar *H. pylori* inside *Candida albicans* were protected from environmental stress and antibiotics such as amoxicillin, and once unfavorable conditions subsided, the *H. pylori* were released. It was noticed as urease activity on urea-based agar and further detected by PCR. The released bacterium was able to facilitate its entry into new target cells and cause inflammation in the gastric environment [24].

The relationship between the *H. pylori* infection and its existence in Candida vacuoles is still not sufficiently known, as well as its importance in resistance and possible use in therapies which is still a field of interest for scientists. However, some studies show that using probiotics might be helpful. Their use can inhibit the virulence of Candida by reducing its adherence or biofilm formation. It was also found that they can have an impact on local and systemic inflammation in organisms infected by Candida. Thus, their addition to therapies for *H. pylori* might be a possible way to fight infections caused by both Candida and *H. pylori* [2,72].

In recent years, there have been ongoing observations of correlations not only between *H. pylori* and *Candida* spp., but also between *H. pylori* and many numerous different microorganisms [73]. One of the best-described examples of such research describes the interactions with Epstein–Barr virus (EBV), which has been implicated in gastric carcinogenesis through its ability to modulate host immune responses and promote oncogenic transformation. The study conducted by Gareayaghi et al. showed a significant difference in a group of patients with gastric cancer. In this study, the presence of the EBER-1 gene, specific to Epstein–Barr virus infection, was significant with an odds ratio of 3.319 [74]. Another study shows that co-existence of *H. pylori* infection and presence of EBV is significantly higher in patients with gastric cancer or peptic ulcer disease than in patients with non-ulcer dyspepsia (NUD), and prevalence of EBV DNA alone is more often found among the patients with those conditions (GC vs. NUD = 90% vs. 37%, *p* < 0.001; PUD vs. NUD = 70% vs. 37%, *p* < 0.001) [75]. The level of the EBV-DNA copies was higher in groups with GC and PU than NUD with significant differences between them [76]. Following the example of these studies, the assumption could be made that the co-occurrence of *Candida* and *H. pylori* seems to be sometimes critical for *H. pylori* survival. At present, the basis of this mechanism is simply the structure of the fungus and the structures that prevent the antibiotic from penetrating into the vacuole structures with hidden bacteria, but further research is needed.

## 8. Conclusions

*H. pylori*, like *Candida* itself, is a very common finding in the human body, which is why their common existence often overlaps. This creates a situation in which both organisms must coexist, which leaves us with the question of the meaning of such a condition and the clinical implications for humans.

The theory presented in this paper on the association between *Candida* and *H. pylori* co-occurrence and recurrent gastroenterological symptoms with concomitant unsuccessful eradication requires further studies and clinical trials to directly demonstrate the impact on prevalence and the design of therapies that take into account the presence of the fungus.

Given the growing problem of recurrent infections after apparently successful treatment, and the resulting serious complications, representing not only a direct burden on the patient’s health but also a financial cost to the health service, consideration of therapeutic steps to limit this phenomenon should be considered one of the priorities in the treatment and follow-up of gastroenterological patients.

## Figures and Tables

**Table 2:** Worldwide estimated prevalence of *H. pylori* antibiotic resistance [53].

Region	Worldwide Prevalence of *H. pylori* Antibiotic Resistance (%)				
	Clarithromycin	Metronidazole	Levofloxacin	Amoxicillin	Tetracycline
**Africa**	15	91	14	38	13
**Americas**	14	27	14	8	4
**Eastern Mediterranean region**	29	61	23	14	10
**European region**	32	38	14	0	0
**Southeast Asia**	17	59	25	12	0
**Western Pacific region**	34	55	24	1	2

## References

[1] Siavoshi F., Saniee P. (2014). Vacuoles of Candida Yeast as a Specialized Niche for *Helicobacter pylori*. World J. Gastroenterol..

[2] Hui W.W., Emerson L.E., Clapp B., Sheppe A.E., Sharma J., del Castillo J., Ou M., Maegawa G.H.B., Hoffman C., Larkin J. (2021). The Cross-Kingdom Interaction between *Helicobacter pylori* and Candida Albicans. PLoS Pathog..

[3] Ali A., AlHussaini K.I. (2024). *Helicobacter pylori*: A Contemporary Perspective on Pathogenesis, Diagnosis and Treatment Strategies. Microorganisms.

[4] Kesharwani A., Dighe O.R., Lamture Y. (2023). Role of *Helicobacter pylori* in Gastric Carcinoma: A Review. Cureus.

[5] Baj J., Forma A., Sitarz M., Portincasa P., Garruti G., Krasowska D., Maciejewski R. (2020). *Helicobacter pylori* Virulence Factors—Mechanisms of Bacterial Pathogenicity in the Gastric Microenvironment. Cells.

[6] Azevedo N.F., Huntington J., Goodman K.J. (2009). The Epidemiology of *Helicobacter pylori* and Public Health Implications. Helicobacter.

[7] Sachs G., Scott D.R. (2012). *Helicobacter pylori*: Eradication or Preservation. F1000 Med. Rep..

[8] Salvatori S., Marafini I., Laudisi F., Monteleone G., Stolfi C. (2023). *Helicobacter pylori* and Gastric Cancer: Pathogenetic Mechanisms. Int. J. Mol. Sci..

[9] Goni E., Franceschi F. (2016). *Helicobacter pylori* and Extragastric Diseases. Helicobacter.

[10] Maiorana F., Neschuk M., Caronia M.V., Elizondo K., Robledo M.L., Schneider A., Veron G., Zapata P.D., Barreyro F.J. (2024). The Interplay between *Helicobacter pylori* Infection and Rs738409 PNPLA3 in Metabolic Dysfunction-Associated Steatotic Liver Disease. PLoS ONE.

[11] Keikha M., Karbalaei M. (2022). Potential Association between Bacterial Infections and Ischemic Stroke Based on Fifty Case-Control Studies: A Systematic Review and Meta-Analysis. New Microbes New Infect..

[12] Keikha M., Karbalaei M. (2022). A Comprehensive Survey of the Relationship between *Helicobacter pylori* Infection and Atherosclerosis in the Iranian Population: A Systematic Review and Meta-Analysis. Arch. Iran Med..

[13] Álvarez-Arellano L. (2014). *Helicobacter pylori* and Neurological Diseases: Married by the Laws of Inflammation. World J. Gastrointest. Pathophysiol..

[14] Kusters J.G., Van Vliet A.H.M., Kuipers E.J. (2006). Pathogenesis of *Helicobacter pylori* Infection. Clin. Microbiol. Rev..

[15] Kao C.Y., Sheu B.S., Wu J.J. (2016). *Helicobacter pylori* Infection: An Overview of Bacterial Virulence Factors and Pathogenesis. Biomed. J..

[16] Yang T., Li J., Zhang Y., Deng Z., Cui G., Yuan J., Sun J., Wu X., Hua D., Xiang S. (2024). Intracellular Presence of *Helicobacter pylori* Antigen and Genes within Gastric and Vaginal Candida. PLoS ONE.

[17] Zou Y., Chen X., Sun Y., Li P., Xu M., Fang P., Zhang S., Yuan G., Deng X., Hu H. (2022). Antibiotics-Free Nanoparticles Eradicate *Helicobacter pylori* Biofilms and Intracellular Bacteria. J. Control. Release.

[18] Sánchez-Alonzo K., Matamala-Valdés L., Parra-Sepúlveda C., Bernasconi H., Campos V.L., Smith C.T., Sáez K., García-Cancino A. (2021). Intracellular Presence of *Helicobacter pylori* and Its Virulence-Associated Genotypes within the Vaginal Yeast of Term Pregnant Women. Microorganisms.

[19] Tang Z., Fu L., Liu R., Chen Y., Bie M., Wang B. (2023). Mechanisms of intracellular *Helicobacter pylori* infection and clinical considerations. J. Sichuan Univ..

[20] Huang Y., Wang Q.L., Cheng D.D., Xu W.T., Lu N.H. (2016). Adhesion and Invasion of Gastric Mucosa Epithelial Cells by *Helicobacter pylori*. Front. Cell. Infect. Microbiol..

[21] McBain A.J., O’Neill C.A., Oates A. (2016). Skin Microbiology. Reference Module in Biomedical Sciences.

[22] Dowd F.J. (2014). Candida Albicans Infections. Reference Module in Biomedical Research.

[23] Wiles C.M., Mackenzie D.W.R. (1987). Fungal Diseases of the Central Nervous System. Infect. Nerv. Syst..

[24] Hiengrach P., Panpetch W., Chindamporn A., Leelahavanichkul A. (2022). *Helicobacter pylori*, Protected from Antibiotics and Stresses Inside Candida Albicans Vacuoles, Cause Gastritis in Mice. Int. J. Mol. Sci..

[25] (2001). The ALS Gene Family of Candida Albicans. Trends Microbiol..

[26] Naglik J.R., Challacombe S.J., Hube B. (2003). Candida Albicans Secreted Aspartyl Proteinases in Virulence and Pathogenesis. Microbiol. Mol. Biol. Rev..

[27] Gaffen S.L., Hernández-Santos N., Peterson A.C. (2011). IL-17 Signaling in Host Defense Against Candida Albicans. Immunol. Res..

[28] Borka Balas R., Meliț L.E., Mărginean C.O. (2022). Worldwide Prevalence and Risk Factors of *Helicobacter pylori* Infection in Children. Children.

[29] Tran V., Saad T., Tesfaye M., Walelign S., Wordofa M., Abera D., Desta K., Tsegaye A., Ay A., Taye B. (2022). *Helicobacter pylori* (*H. pylori*) Risk Factor Analysis and Prevalence Prediction: A Machine Learning-Based Approach. BMC Infect. Dis..

[30] Smith S., Jolaiya T., Fowora M., Palamides P., Ngoka F., Bamidele M., Lesi O., Onyekwere C., Ugiagbe R., Agbo I. (2018). Clinical and Socio- Demographic Risk Factors for Acquisition of *Helicobacter pylori* Infection in Nigeria. Asian Pac. J. Cancer Prev..

[31] Chen Y.C., Malfertheiner P., Yu H.T., Kuo C.L., Chang Y.Y., Meng F.T., Wu Y.X., Hsiao J.L., Chen M.J., Lin K.P. (2024). Global Prevalence of *Helicobacter pylori* Infection and Incidence of Gastric Cancer Between 1980 and 2022. Gastroenterology.

[32] Ernst P.B., Gold B.D. (2000). The Disease Spectrum of *Helicobacter pylori*: The Immunopathogenesis of Gastroduodenal Ulcer and Gastric Cancer. Annu. Rev. Microbiol..

[33] McConaghy J.R., Decker A., Nair S. (2023). Peptic Ulcer Disease and *H. pylori* Infection: Common Questions and Answers. Am. Fam. Physician.

[34] Chen T.H., Cheng H.T., Yeh C.T. (2021). Epidemiology Changes in Peptic Ulcer Diseases 18 Years Apart Explored from the Genetic Aspects of *Helicobacter pylori*. Transl. Res..

[35] Jia Z., Zheng M., Jiang J., Cao D., Wu Y., Zhang Y., Fu Y., Cao X. (2022). Positive *H. pylori* Status Predicts Better Prognosis of Non-Cardiac Gastric Cancer Patients: Results from Cohort Study and Meta-Analysis. BMC Cancer.

[36] He J.J., Hu W.C., Ouyang Q., Zhang S.W., He L.J., Chen W.Y., Li X.Z., Hu C.J. (2022). *Helicobacter pylori* Infection Induces Stem Cell-like Properties in Correa Cascade of Gastric Cancer. Cancer Lett..

[37] Gu Y., Xu Y., Wang P., Zhao Y., Wan C. (2024). Research Progress on Molecular Mechanism of Pyroptosis Caused by *Helicobacter pylori* in Gastric Cancer. Ann. Med. Surg..

[38] Kumar S., Metz D.C., Ellenberg S., Kaplan D.E., Goldberg D.S. (2020). Risk Factors and Incidence of Gastric Cancer After Detection of *Helicobacter pylori* Infection: A Large Cohort Study. Gastroenterology.

[39] Watari J., Das K.K., Amenta P.S., Tanabe H., Tanaka A., Geng X., Lin J.J.C., Kohgo Y., Das K.M. (2008). Effect of Eradication of *Helicobacter pylori* on the Histology and Cellular Phenotype of Gastric Intestinal Metaplasia. Clin. Gastroenterol. Hepatol..

[40] Pimanov S.I., Makarenko E.V., Voropaeva A.V., Matveenko M.E., Voropaev E.V. (2008). *Helicobacter pylori* Eradication Improves Gastric Histology and Decreases Serum Gastrin, Pepsinogen I and Pepsinogen II Levels in Patients with Duodenal Ulcer. J. Gastroenterol. Hepatol..

[41] Rokkas T., Pistiolas D., Sechopoulos P., Robotis I., Margantinis G. (2007). The Long-Term Impact of *Helicobacter pylori* Eradication on Gastric Histology: A Systematic Review and Meta-Analysis. Helicobacter.

[42] Li W.Q., Ma J.L., Zhang L., Brown L.M., Li J.Y., Shen L., Pan K.F., Liu W.D., Hu Y., Han Z.X. (2014). Effects of *Helicobacter pylori* Treatment on Gastric Cancer Incidence and Mortality in Subgroups. J. Natl. Cancer Inst..

[43] Gisbert J.P., McNicholl A.G. (2017). Optimization Strategies Aimed to Increase the Efficacy of *H. pylori* Eradication Therapies. Helicobacter.

[44] Malfertheiner P., Megraud F., Rokkas T., Gisbert J.P., Liou J.-M., Schulz C., Gasbarrini A., Hunt R.H. (2022). Management of *Helicobacter pylori* Infection: The Maastricht VI/Florence Consensus Report. Gut.

[45] Salazar C.O., Cardenas V.M., Reddy R.K., Dominguez D.C., Snyder L.K., Graham D.Y. (2012). Greater than 95% Success with 14-Day Bismuth Quadruple Anti- *Helicobacter pylori* Therapy: A Pilot Study in US Hispanics. Helicobacter.

[46] Yuan Y., Ford A.C., Khan K.J., Gisbert J.P., Forman D., Leontiadis G.I., Tse F., Calvet X., Fallone C., Fischbach L. (2013). Optimum Duration of Regimens for *Helicobacter pylori* Eradication. Cochrane Database Syst. Rev..

[47] Thung I., Aramin H., Vavinskaya V., Gupta S., Park J.Y., Crowe S.E., Valasek M.A. (2016). Review Article: The Global Emergence of *Helicobacter pylori* Antibiotic Resistance. Aliment. Pharmacol. Ther..

[48] ACG American College of Gastroenterology, Guideline on Treatment of *Helicobacter pylori*: New Recommendations… Will Practice Change?. https://gi.org/journals-publications/ebgi/schoenfeld_sep2024/.

[49] McNicholl A.G., Linares P.M., Nyssen O.P., Calvet X., Gisbert J.P. (2012). Meta-Analysis: Esomeprazole or Rabeprazole vs. First-Generation Pump Inhibitors in the Treatment of *Helicobacter pylori* Infection. Aliment. Pharmacol. Ther..

[50] Gong Y., Yuan Y. (2018). Resistance Mechanisms of *Helicobacter pylori* and Its Dual Target Precise Therapy. Crit. Rev. Microbiol..

[51] Tshibangu-Kabamba E., Yamaoka Y. (2021). *Helicobacter pylori* Infection and Antibiotic Resistance—from Biology to Clinical Implications. Nat. Rev. Gastroenterol. Hepatol..

[52] Savoldi A., Carrara E., Graham D.Y., Conti M., Tacconelli E. (2018). Prevalence of Antibiotic Resistance in *Helicobacter pylori*: A Systematic Review and Meta-Analysis in World Health Organization Regions. Gastroenterology.

[53] Salahi-Niri A., Nabavi-Rad A., Monaghan T.M., Rokkas T., Doulberis M., Sadeghi A., Zali M.R., Yamaoka Y., Tacconelli E., Yadegar A. (2024). Global Prevalence of *Helicobacter pylori* Antibiotic Resistance among Children in the World Health Organization Regions between 2000 and 2023: A Systematic Review and Meta-Analysis. BMC Med..

[54] Katzenstein A.L.A., Maksem J. (1979). Candidal Infection of Gastric Ulcers. Histology, Incidence, and Clinical Significance. Am. J. Clin. Pathol..

[55] Scott B.B., Jenkins D. (1982). Gastro-Oesophageal Candidiasis. Gut.

[56] Robin Warren J., Marshall B. (1983). Unidentified Curved Bacilli On Gastric Epithelium In Active Chronic Gastritis. Lancet.

[57] Zwolińska-Wcisło M., Budak A., Trojanowska D., Bogdał J., Stachura J. (1998). Fungal Colonization of the Stomach and Its Clinical Relevance. Mycoses.

[58] Medical Science Monitor, Effect of Fungal Colonization of Gastric Mucosa on the Course of Gastric Ulcers Healing. Article Abstract #421150. https://medscimonit.com/abstract/index/idArt/421150.

[59] PubMed, Assessment of Co-Existence of *Helicobacter pylori* and Candida Fungi in Diseases of the Upper Gastrointestinal Tract. https://pubmed.ncbi.nlm.nih.gov/20224149/.

[60] Wang Y.H., Lv Z.F., Zhong Y., Liu D.S., Chen S.P., Xie Y. (2017). The Internalization of *Helicobacter pylori* Plays a Role in the Failure of *H. pylori* Eradication. Helicobacter.

[61] D’Enfert C., Kaune A.K., Alaban L.R., Chakraborty S., Cole N., Delavy M., Kosmala D., Marsaux B., Fróis-Martins R., Morelli M. (2021). The Impact of the Fungus-Host-Microbiota Interplay upon Candida Albicans Infections: Current Knowledge and New Perspectives. FEMS Microbiol. Rev..

[62] Heydari S., Siavoshi F., Jazayeri M.H., Sarrafnejad A., Saniee P. (2020). *Helicobacter pylori* Release from Yeast as a Vesicle-Encased or Free Bacterium. Helicobacter.

[63] Plant cell biology, Volume 1, PWN. https://ksiegarnia.pwn.pl/Biologia-komorki-roslinnej-Tom-1-Struktura,68710268,p.html?srsltid=AfmBOopJzuyr-7vIiLAziGblmX-O8gomgJuV5XVfUzCd4WosnmEx6E7J.

[64] Siavoshi F., Heydari S., Shafiee M., Ahmadi S., Saniee P., Sarrafnejad A., Kolahdoozan S. (2019). Sequestration inside the Yeast Vacuole May Enhance *Helicobacter pylori* Survival against Stressful Condition. Infect. Genet. Evol..

[65] Scott D.R., Sachs G., Marcus E.A. (2016). The Role of Acid Inhibition in *Helicobacter pylori* Eradication. F1000Research.

[66] Yang J.C., Lu C.W., Lin C.J. (2014). Treatment of *Helicobacter pylori* Infection: Current Status and Future Concepts. World J. Gastroenterol..

[67] Siavoshi F., Sahraee M., Ebrahimi H., Sarrafnejad A., Saniee P. (2018). Natural Fruits, Flowers, Honey, and Honeybees Harbor *Helicobacter pylori*-Positive Yeasts. Helicobacter.

[68] Sánchez-Alonzo K., Silva-Mieres F., Arellano-Arriagada L., Parra-Sepúlveda C., Bernasconi H., Smith C.T., Campos V.L., García-Cancino A. (2021). Nutrient Deficiency Promotes the Entry of *Helicobacter pylori* Cells into Candida Yeast Cells. Biology.

[69] Sánchez-Alonzo K., Parra-Sepúlveda C., Vega S., Bernasconi H., Campos V.L., Smith C.T., Sáez K., García-Cancino A. (2020). In Vitro Incorporation of *Helicobacter pylori* into Candida Albicans Caused by Acidic PH Stress. Pathogens.

[70] Sánchez-Alonzo K., Belmar L., Parra-Sepúlveda C., Bernasconi H., Campos V.L., Smith C.T., Sáez K., García-Cancino A. (2021). Antibiotics as a Stressing Factor Triggering the Harboring of *Helicobacter pylori* J99 within Candida Albicans ATCC10231. Pathogens.

[71] Hildebrandt E., McGee D.J. (2009). *Helicobacter pylori* Lipopolysaccharide Modification, Lewis Antigen Expression, and Gastric Colonization Are Cholesterol-Dependent. BMC Microbiol..

[72] Ribeiro F.C., Rossoni R.D., de Barros P.P., Santos J.D., Fugisaki L.R.O., Leão M.P.V., Junqueira J.C. (2020). Action Mechanisms of Probiotics on Candida Spp. and Candidiasis Prevention: An Update. J. Appl. Microbiol..

[73] Fan Y., Chen X., Shan T., Wang N., Han Q., Ren B., Cheng L. (2025). Polymicrobial Interactions of *Helicobacter pylori* and Its Role in the Process of Oral Diseases. J. Oral Microbiol..

[74] Gareayaghi N., Akkus S., Saribas S., Demiryas S., Ozbey D., Kepil N., Demirci M., Dinc H.O., Akcin R., Uysal O. (2021). Epstein-Barr Virus and *Helicobacter pylori* Co-Infection in Patients with Gastric Cancer and Duodenale Ulcer. New Microbiol..

[75] Shukla S.K., Prasad K.N., Tripathi A., Singh A., Saxena A., Chand Ghoshal U., Krishnani N., Husain N., Prasad K.N. (2011). Epstein-Barr Virus DNA Load and Its Association with *Helicobacter pylori* Infection in Gastroduodenal Diseases. Braz. J. Infect. Dis..

[76] Akkus S., Gareayaghi N., Saribas S., Demiryas S., Ozbey D., Kepil N., Demirci M., Ziver Sarp T., Oyku Dinc H., Akcin R. (2022). Co-Infection Relationship with Epstein-Barr Virus in Gastroduodenal Diseases with *Helicobacter pylori*. Quantitative PCR and EBNA-1 Gene-Based Approach. Acta Gastroenterol. Belg..

